# Comfort Terminal Care auf der Intensivstation: Empfehlungen für die Praxis

**DOI:** 10.1007/s00101-024-01382-9

**Published:** 2024-02-05

**Authors:** Eva Schaden, Helga Dier, Dietmar Weixler, Walter Hasibeder, Andrea Lenhart-Orator, Christian Roden, Sonja Fruhwald, Barbara Friesenecker, ARGE Ethik in Anästhesie und Intensivmedizin der ÖGARI

**Affiliations:** 1 Universitätsklinik für Anästhesie, Allgemeine Intensivmedizin und Schmerztherapie, Medizinische Universität Wien, Währinger Gürtel 18–20, 1090 Wien, Österreich; 2 Klinische Abteilung für Anästhesie und Intensivmedizin, Universitätsklinikum St. Pölten, St. Pölten, Österreich; 3 Palliativkonsiliardienst und mobiles Palliativteam, Landesklinikum Horn-Allentsteig, Horn, Österreich; 4 Abteilung für Anästhesie und Perioperative Intensivmedizin, St. Vinzenz Krankenhaus Betriebs GmbH Zams, Zams, Österreich; 5 Abteilung für Anästhesie, Intensiv-, und Schmerzmedizin, Klinik Ottakring Wien; i.R., Wien, Österreich; 6 Anästhesie und Intensivmedizin, Palliativstation, Krankenhaus der Barmherzigen Schwestern Ried, Ried im Innkreis, Österreich; 7 Klinische Abteilung für Anästhesiologie und Intensivmedizin 2, Universitätsklinik für Anästhesiologie und Intensivmedizin, Medizinische Universität Graz, Graz, Österreich; 8 Universitätsklinik für Allgemeine und Chirurgische Intensivmedizin, Medizinische Universität Innsbruck, Innsbruck, Österreich

**Keywords:** Ethik, Intensivmedizin, Palliativmedizin, Therapiezieländerung, Terminal Care, Ethics, Critical care, Palliative care, Patient-centered care, Terminal care

## Abstract

**Hintergrund und Ziel der Arbeit:**

Die Arbeitsgemeinschaft Ethik in Anästhesie und Intensivmedizin der Österreichischen Gesellschaft für Anästhesiologie, Reanimation und Intensivmedizin (ÖGARI) hat schon vor 10 Jahren Dokumentationswerkzeuge für die Therapiezieländerung entwickelt. Seither hat insbesondere die praktische Umsetzung der Comfort Terminal Care in der täglichen Routine zahlreiche Fragenstellungen aufgeworfen, die in dieser Folgearbeit diskutiert und, wann immer möglich, evidenzbasiert beantwortet werden.

**Ergebnisse:**

Die praktische Umsetzung der Schmerztherapie sowie der Reduktion von Angst, Stress und Atemnot, die im Rahmen einer Comfort Terminal Care selbstverständlich indiziert sind, werden näher beschrieben. Zu den Maßnahmen, die nicht (mehr) indiziert sind, wie Sauerstoffgabe und Beatmung sowie Gabe von Flüssigkeit und Ernährung, wird Stellung genommen. Weiterhin werden Empfehlungen hinsichtlich Monitoring, (Labor‑)Befunderhebung sowie zu medikamentösen Therapien gegeben und die Bedeutung von Pflegehandlungen im Rahmen der Comfort Terminal Care benannt. Zuletzt werden noch die Begleitung der An- und Zugehörigen und das Vorgehen in der Zeit nach dem Versterben dargestellt.

**Diskussion:**

Eine Therapiezieländerung mit zeitgerechter Umstellung auf Comfort Terminal Care ermöglicht eine gute und menschliche Betreuung schwerst kranker Patient:innen und ihrer An- und Zugehörigen am Lebensende und die Wertschätzung ihres bisherigen Lebens mit der Möglichkeit positiver Erfahrungen bis zuletzt.

## Abstract (en)

**Background and objective:**

The Working Group on Ethics in Anesthesia and Intensive Care Medicine of the Austrian Society for Anesthesiology Resuscitation and Intensive Care Medicine (ÖGARI) already developed documentation tools for the adaption of therapeutic goals 10 years ago. Since then the practical implementation of Comfort Terminal Care in the daily routine in particular has raised numerous questions, which are discussed in this follow-up paper and answered in an evidence-based manner whenever possible.

**Results:**

The practical implementation of pain therapy and reduction of anxiety, stress and respiratory distress that are indicated in the context of Comfort Terminal Care are described in more detail. The measures that are not (or no longer) indicated, such as oxygen administration and ventilation as well as the administration of fluids and nutrition, are also commented on. Furthermore, recommendations are given regarding monitoring, (laboratory) findings and drug treatment and the importance of nursing actions in the context of Comfort Terminal Care is mentioned. Finally, the support for the next of kin and the procedure in the time after death are presented.

**Discussion:**

A change in treatment goals with a timely switch to Comfort Terminal Care enables good and humane care for seriously ill patients and their relatives at the end of life and the appreciation of their previous life with the possibility of positive experiences until the end.

## Einleitung

Comfort Terminal Care (CTC; Komforttherapie) beschreibt die ganzheitliche Betreuung schwerst kranker Patient:innen in der letzten Phase ihres Lebens. Das ursprünglich festgelegte Therapieziel Heilung resp. Besserung wird verlassen, und es wird – falls möglich gemeinsam mit dem:der Patient:in – eine Therapiezieländerung (TZÄ) [1, 2] in Richtung Palliation [3, 4] festgelegt. Belastende Interventionen werden fortan vermieden, denn das Hauptaugenmerk aller therapeutischen Handlungen und Interventionen liegt nun auf dem Erreichen einer bestmöglichen Lebensqualität mit größtmöglichem Wohlbefinden (Comfort) durch eine möglichst gute Symptomlinderung. So wird – bezugnehmend auf die Stellungnahme der Bioethikkommission des österreichischen Bundeskanzleramtes [5] – ein Sterben in Würde ermöglicht. Wird im Rahmen der CTC eine definierte Therapie unterlassen, entspricht dies einer rechtlich zulässigen Nichtdurchführung („withhold“ – wenn die Indikation für den Beginn einer Behandlung primär nicht gegeben ist) oder einer ebenfalls rechtlich zulässigen Nichtweiterführung/Beendigung („withdraw“ – wenn die Indikation für die Weiterführung einer Behandlung nicht (mehr) gegeben ist). Der „Beistand beim Sterben“ ist somit nicht nur ethisch gefordert, sondern auch rechtlich geboten (§ 49a Österreichisches Ärztegesetz [6]).

Der Entscheidung zur CTC sollte eine intensive, interdisziplinäre Diskussion über die Indikation, den individuellen Nutzen und die Belastungen von technisch machbaren Therapien auf Basis einer Prognoseeinschätzung hinsichtlich Überlebenswahrscheinlichkeit und zukünftiger Funktionalität vorausgehen. Jedes Teammitglied (Ärzt:innen, Pflegepersonen, Physiotherapeut:innen, Psycholog:innen …) sollte, so gewünscht, im Entscheidungsprozess der Therapiezieländerung angehört werden. Grundsätzlich gilt: Lebensqualität vor Lebenszeit! Spätestens in dieser Phase der Entscheidungsfindung sollten ausführliche Gespräche mit dem:der Patient:in selbst und – falls von dem:der Patient:in (mutmaßlich) gewünscht – mit den An- und Zugehörigen/Vertrauenspersonen geführt werden. In Österreich haben An- und Zugehörige – sofern sie nicht die Erwachsenenvertretung oder Vorsorgevollmacht für eine:n Patient:in übernommen haben – keine Rechtsstellung in Bezug auf medizinische Entscheidungen. Sie sind aber wichtige Kommunikationspartner:innen im oft schwierigen Entscheidungsprozess rund um eine TZÄ, denn sie können Entscheidungen erleichtern, indem sie den mutmaßlichen Patient:innenwillen vermitteln. Gespräche im Sinne eines „Advance Care Planning“ (ACP) sollten bereits im Vorfeld von großen Operationen, Chemo-, und Strahlentherapien erfolgen. Hierzu benötigt es wiederholte Gespräche, zu denen schwerst kranke Patient:innen meist nicht (mehr) in der Lage sind. Im Rahmen des ACP sollte auch noch die Erstellung einer Patient:innenverfügung und/oder einer Vorsorgevollmacht in Erwägung gezogen werden bzw. zumindest eine Person des Vertrauens benannt werden.

Die letztendliche Verantwortung für die Entscheidung zur Therapiezieländerung mit Umstellung auf CTC trägt immer der:die für den:die Patient:in verantwortliche Fachärzt:in. Sollte initial kein für alle Beteiligten akzeptabler Konsens erreicht werden, gilt „in dubio pro vita“, und die Therapie wird zunächst fortgesetzt, bis eine einstimmige Entscheidung gefällt werden kann. Hierfür hat sich das Konzept des Time-Limited Trial (TLT) [7, 8] bewährt. Ein TLT ist eine Vereinbarung zwischen dem Behandlungsteam und eine:r Patient:in bzw. den An- und Zugehörigen, bestimmte Therapien über einen bestimmten Zeitraum anzuwenden, um zu sehen, wie sich der Zustand des:der Patient:in im Hinblick auf die vereinbarten Therapieziele entwickelt. Wenn sich der:die Patient:in verbessert, wird die Therapie fortgesetzt. Verschlechtert sich der Zustand des:der Patient:in, werden die im TLT überprüften Therapien abgesetzt und i. d. R. palliative Therapieziele vereinbart. Während eines TLT sollte der:die Patient:in insbesondere klinisch engmaschig überwacht werden, um zeitgerechtes Handeln zu ermöglichen, wenn der schmale Grat der Chance auf Verbesserung verlassen wird und es zu einer Verlängerung des Leidens kommt [9].

Alle Handlungen im Rahmen einer TZÄ und CTC müssen genauso exakt dokumentiert werden, wie jegliche auf Heilung ausgerichtete Therapie. Die Arbeitsgemeinschaft Ethik in Anästhesie und Intensivmedizin der Österreichischen Gesellschaft für Anästhesiologie, Reanimation und Intensivmedizin (ÖGARI) hat schon vor 10 Jahren Dokumentationswerkzeuge für die TZÄ entwickelt, die in österreichischen Krankenanstalten in zunehmenden Maß Verwendung finden [1].

Seither hat aber insbesondere die praktische Umsetzung der CTC in der täglichen Routine zahlreiche Fragenstellungen aufgeworfen, die nun im Rahmen dieser Folgearbeit diskutiert und, wann immer möglich, evidenzbasiert beantwortet werden. Es werden Schmerztherapie sowie Reduktion von Angst, Stress und Atemnot, die im Rahmen einer CTC selbstverständlich indiziert sind, näher beschrieben. Zu den Maßnahmen, die nicht (mehr) indiziert sind, wie Sauerstoffgabe und Beatmung sowie Gabe von Flüssigkeit und Ernährung, wird Stellung genommen. Weiterhin werden Empfehlungen hinsichtlich Monitoring, (Labor‑)Befunderhebung sowie zu medikamentösen Therapien gegeben und die Bedeutung von Pflegehandlungen im Rahmen der CTC benannt. Zuletzt werden noch die Begleitung der An- und Zugehörigen und das Vorgehen in der Zeit nach dem Versterben dargestellt.

## Maßnahmen

**Figure 1:**
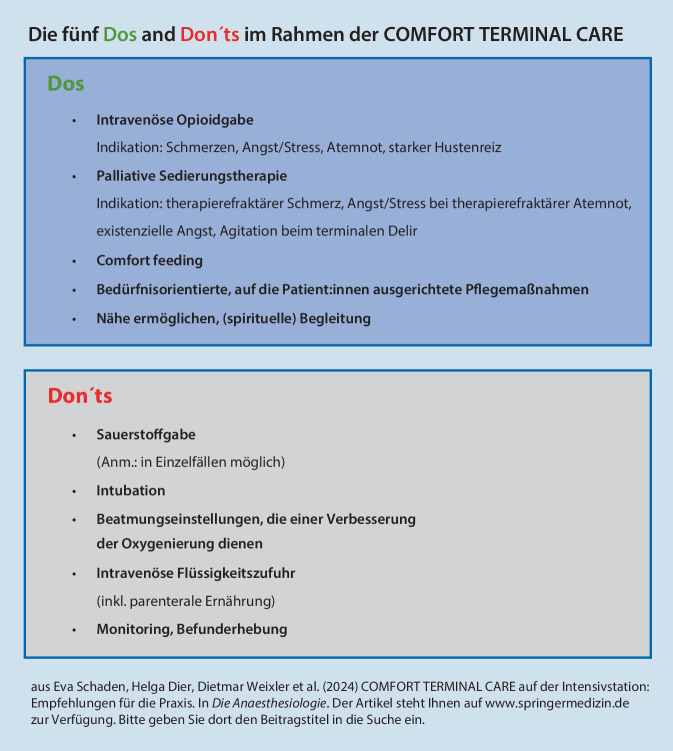

In der praktischen Umsetzung der CTC ist es ist von großer Wichtigkeit, alle Teammitglieder einschließlich der An- und Zugehörigen in den Prozess miteinzubinden. Dabei soll in Erinnerung gerufen werden, dass im Rahmen der CTC laufende Therapien, die den Sterbeprozess hinauszögern und Leiden verlängern, beendet und stattdessen Maßnahmen, die für den Patient:innenkomfort in der Sterbephase wichtig sind, den Symptomen entsprechend angepasst/gesteigert werden. Treten bei Patient:innen und/oder An- und Zugehörigen Symptome auf, die mithilfe der alleinigen Expertise der Intensivteams nicht behandelt werden können, sollten unbedingt Teams der spezialisierten Palliativversorgung eingeschaltet werden und in die Behandlung fortlaufend integriert werden. Hier hat sich auch der „Mixed Approach“ von intensivmedizinischen und Palliative Care Teams in der Betreuung sterbender Intensivpatient:innen bewährt [10]. Die einzelnen Maßnahmen im Rahmen der CTC werden im Folgenden detailliert ausgeführt; einen Überblick bietet Abb. 1.

### Schmerzen

Nicht nur in der Sterbephase stellen Schmerzen für viele Menschen die größte Belastung dar. Schmerzen unterliegen einer subjektiven Wahrnehmung; wenn Patient:innen Schmerzen angeben, haben sie also recht. Allem voran sollte eine Diagnostik in Bezug auf Schmerz durchgeführt werden, um auch nichtoffensichtliche Schmerzursachen zu erfassen und solche zu erkennen, die ohne Medikamente z. B. durch Lagerungsbehelfe gelindert werden können. Optimales Schmerzmanagement setzt die Verwendung von Instrumenten voraus, die eine klinische Schmerzbeurteilung ermöglichen. Dazu zählen z. B. die „Behavioral Pain Scale (BPS)“ [11] oder das „Critical Care Pain Observation Tool“ [12]. Die Dosierung von Analgetika und insbesondere Opioiden hat sich an der von dem:der Patient:in angegebenen und nach klinischen Kriterien eingeschätzten Schmerzintensität zu orientieren. In einer Untersuchung bei Intensivpatient:innen mit CTC berichtet die Arbeitsgruppe um Hall erhebliche Variationen der in den letzten 12 Lebensstunden verabreichten Morphindosen (durchschnittlich verabreichte Morphindosis/12 Stunden: 32 Milligramm [mg]; Range: 1–698 mg) [13]. Ähnliches zeigt eine kanadische Multizenterstudie, in der Morphindosen in den letzten 4 Stunden im Rahmen der Rücknahme mechanischer Beatmung zwischen 2 und 450 mg variierten [14].

### Angst, Stress

Schwer kranke Patient:innen erleiden häufig existenzielle Ängste. Als Mittel der ersten Wahl gegen Angst gilt menschlicher Beistand. Zu den Basismaßnahmen, um Angst bei sterbenden Patient:innen zu reduzieren, gehört auch eine beruhigte Patient:innenumgebung, die eine Atmosphäre der Sicherheit und Geborgenheit schafft. Insbesondere auf Intensivstationen ist dieses Ziel oft schwer erreichbar. Dennoch sollte für sterbende Patient:innen durch deutliche Reduktion oder Beenden des Monitorings (Alarme!, s. unten) und räumliche Abschirmung (z. B. Paravents) ein Ort der Ruhe geschaffen werden. Gleichzeitig ist aber auch eine erweiterte Besuchsmöglichkeit für An- und Zugehörige außerhalb bestehender Besuchszeiten wichtig. Wahrhafte und empathische Kommunikation mit dem:der Patient:in und den An- und Zugehörigen, eine spirituelle und/oder psychologische Begleitung sowie das Vorhandensein und Erleben persönlicher Gegenstände, Musik und Lieblingsdüfte sind weitere wichtige Elemente der Angstprävention und -therapie.

Bei wachen, kooperativen Patient:innen können Ängste durch gezieltes Nachfragen und Selbsteinschätzung erfasst und z. B. nach einer „Numeric Rating Scale“ (NRS; 0 = keine Angst, 10 = schlimmste vorstellbare Angst) quantifiziert werden. Besonders bei bewusstseinsgetrübten und/oder beatmeten Patient:innen ist es schwierig, Ängste von Schmerzreaktionen und/oder Symptomen eines (hyperaktiven) Delirs abzugrenzen. Unruhe und Delir sind häufige neurologische Syndrome unterschiedlicher Genese in der Sterbephase, die nicht nur für den:die Sterbende:n, sondern auch für An- und Zugehörige und das Behandlungsteam eine erhebliche Belastung darstellen. Der routinemäßige Einsatz von Sedierungsskalen, wie z. B. der „Richmond Agitation and Sedation Scale“ (RASS) [15] oder der für Palliativpatient:innen modifizierten Version [16] ist hier sinnvoll.

Ziel der Behandlung von Ängsten ist es, Patient:innen in einen entspannten Gemütszustand zurückzuführen. Ist dies unter gleichzeitig weitgehender Erhaltung des Bewusstseins (z. B. RASS −1 bis +2) nicht möglich, wird im Rahmen der CTC auch ein tieferer Sedierungsgrad als Therapieziel angestrebt.

#### Palliative Sedierungstherapie

Als Palliative Sedierungstherapie (PST) bezeichnet man den Einsatz sedierend wirkender Medikamente mit dem Ziel, durch eine Bewusstseinsminderung unerträgliches Leiden, bei sonst therapierefraktären Symptomen, zu lindern. Sie bedarf, wenn möglich, der Zustimmung des:der entscheidungsfähigen Patient:in oder der gesetzlichen Vertretung. Der Begriff „Terminale Sedierung“ ist obsolet, da er unklar und hinsichtlich der Intention missverständlich ist. Bei der PST wird ein Medikament gewählt, das in geringstmöglicher Dosis, die wirksam ist, um durch Bewusstseinsdämpfung das subjektiv (!) erlebte Leiden zu kontrollieren, verabreicht wird. In Deutschland wurde dafür kürzlich der Begriff der „gezielten Sedierung“, definiert als „Sedieren, bei dem der verringerte Bewusstseinszustand das geplante Mittel zum Erreichen des zuvor gefassten Ziels ist“ [17], eingeführt. Die für die Symptomlinderung notwendigen Substanzen werden in kleinen Schritten zum gewünschten Effekt titriert. Europäische Richtlinien wurden von der European Association for Palliative Care (EAPC) [18] und von der Österreichischen Palliativgesellschaft [19] publiziert.

### Atemnot

Im Rahmen eines Intensivaufenthaltes und/oder am Lebensende zählt Dyspnoe zu den häufigsten Stresssymptomen [20]. Dyspnoe ist dabei häufig mit existenziellen Ängsten, Panikattacken und Schlaflosigkeit assoziiert [21, 22]. Dabei verstärken Ängste und Depression das subjektive Erleben von Dyspnoe.

In der Sterbephase finden sich fast immer Symptome, die als Ausdrucksform von Dyspnoe gewertet werden können: Hierzu zählen Tachypnoe, unregelmäßige Atemmuster, Verwendung der Atemhilfsmuskulatur, Rasselatmung, schwacher oder fehlender Hustenstoß und geräuschvolles Ein- und/oder Ausatmen [23]. Atemnot sollte bei bewusstseinsklaren Patient:innen mittels verschiedener Skalen wie der „Numeric Rating Scale“ (0 = keine Atemnot; 10 = schwerste Atemnot), der modifizierten „Borg-Skala“ und der vierstufigen „Verbal Descriptor Scale“ (VDS; keine, milde, moderate, schwere Atemnot) objektiviert werden [24]. Bei bewusstseinsgetrübten Patient:innen wird Dyspnoe oft nur indirekt und unter Ausschluss anderer Ursachen (z. B. Schmerzen, akutes Delir) vermutet. Unspezifische Symptome können Unruhe, Tachypnoe, Einsatz der Atemhilfsmuskulatur, paradoxe Atmung, „Kämpfen gegen die Beatmungsmaschine“, Tachykardie und Hypertension sein. Diese Symptome sind z. B. in der „Respiratory Distress Observation Scale“ (RDOS; Dyspnoe ≥ 3 Punkte [25]) abgebildet. In diesen Situationen bedarf es des Ausschlusses möglicherweise behandelbarer, reversibler pulmonaler Prozesse (z. B. Pleuraergüsse, Bronchospasmus) oder mit der Beatmung assoziierter Faktoren (Obstruktion der oberen Atemwege, Patient:in-Ventilator-Asynchronie) als Ursache der Dyspnoe [25]. Atemnot in der Sterbephase ist nicht mit Sauerstoff (s. unten), sondern mit nichtmedikamentösen Maßnahmen (z. B. Lagerung, Handventilator, …) und mit Opioiden zu behandeln [24, 26]. Die schrittweise Dosissteigerung für eine ausreichende Symptomkontrolle ist analog zur Schmerztherapie schriftlich zu dokumentieren.

### Sauerstoff

Atemnot und Ersticken sind moralisch, gesellschaftlich und ethisch sehr negativ belegt. Atemnot lässt betreuende Ärzt:innen/Pflegepersonen oft unreflektiert zu Sauerstoff, Nasenbrille/Venturi-Maske und u. U. sogar zum Tubus greifen (Stichwort Herzalarm beim Sterbenden), v. a. wenn eine CTC nicht definiert, schriftlich dokumentiert und im Team kommuniziert wurde.

Die Gabe von Sauerstoff wird sehr kritisch betrachtet, denn es gibt keine Korrelation zwischen der subjektiv empfundenen Intensität der Atemnot und der gemessenen Sauerstoffsättigung, und nur Opioide sind in der Lage, die Intensität der Atemnot signifikant zu verringern (s. oben) [24]. Als relevante und unangenehme Nebenwirkung trocknet Sauerstoff die Schleimhäute aus. Zudem werden z. B. Nasenbrillen von manchen Patient:innen als schmerzhafte Fremdkörper empfunden. Sauerstoffmasken können Beengungsgefühle und – insbesondere bei bereits vorgeschädigter Haut (bei z. B. Mangelernährung, schlechter Durchblutungssituation) – Druckstellen verursachen. Durch die Verwendung von Ventilatoren oder leicht auf dem Gesicht sitzenden Masken, über die befeuchtete Raumluft appliziert wird, wird ein Gefühl der „bewegten Luft“ im Gesicht und damit oft eine deutliche Erleichterung erreicht.

### Beatmung

Nichtbeatmete, sterbende Patient:innen sollten nicht mehr intubiert werden [27]. Auch die Neuanlage eines Tracheostomas ist bei sterbenden Patient:innen nicht indiziert. Bei Atemnot sollte wie im Abschn. „Atemnot“ beschrieben vorgegangen werden. Absaugen wird von Patient:innen zumeist als sehr unangenehm empfunden [28], daher wird Schleim o. Ä., wenn überhaupt, nur aus der Mundhöhle abgesaugt.

Bei spontan atmenden, beatmungsunterstützten Patient:innen mit Zeichen der Dyspnoe sollte – wie oben beschrieben – die Sedierung (Opioid ± Benzodiazepine) so lange schrittweise erhöht werden, bis der:die Patient:in ruhig atmet. Die Einstellungen am Respirator dienen nur mehr dem Komfort und nicht mehr einer Verbesserung des Gasaustausches, i. d. R. werden also ein PEEP ≤ 3 mm Hg und eine FiO_2_ von 21 % gewählt. Sofern für den:die Patient:in stressfrei möglich, wird auch die bestehende Unterstützung des einzelnen Atemhubes reduziert. Eine Diskonnexion vom Respirator bei in situ verbleibendem Tubus oder Trachealkanüle und die Versorgung mit einer „künstlichen Nase“ (Heat and Moisture Exchanger, HME) zur Vermeidung von Verborkung und Tubusokklusion sind zu erwägen.

Auch bei nicht spontan atmenden, beatmeten Patient:innen sollten die Beatmungseinstellungen so gewählt werden, dass sie nur dem Komfort und nicht einer Verbesserung des Gasaustausches dienen (palliative Beatmung, z. B. BIPAP mit PEEP von 3 mm Hg, FiO_2_ 21 %, oberes Druckniveau für ~200–300 ml Tidalvolumen einstellen; Atemfrequenz: ~10/min).

Wenn die Fortführung der Beatmung das Sterben hinauszögert und Leiden verlängert, besteht bei spontan atmenden Patient:innen die Möglichkeit einer palliativen Extubation [29, 30]. Eine palliative Extubation muss sorgfältig vorbereitet und unbedingt korrekt durchgeführt werden, um Stresssituationen sowohl für den:die sterbenden Patient:in als auch für die Betreuer:innen und die An- und Zugehörigen zu vermeiden. Der:die Patient:in sollte mit erhöhtem Oberkörper positioniert werden; eine bereits vor der Extubation bestehende Analgosedierung kann vertieft werden [31]. Vor der Entfernung des Tubus kann tracheal abgesaugt werden; nach der Extubation ist das Absaugen von Schleim jedenfalls nicht mehr so leicht möglich, und es kann zum Auftreten von Rasselgeräuschen, der Entwicklung eines Stridors bis hin zur Verlegung des Atemweges kommen.

Wenn der:die Patient:in nicht spontan atmet, ist in den Fällen, in denen die Beatmung das Sterben hinauszögert und Leiden verlängert, auch das „einfache“ Abschalten der Beatmungsmaschine erlaubt. Ein solches Vorgehen wird sowohl von den Betreuer:innen als auch den An- und Zugehörigen oft als sehr belastend empfunden und bedarf, wie auch die palliative Extubation, ausführlicher Vorgespräche und einer gut nachvollziehbaren Dokumentation.

Analog bedarf das Abschalten einer ECMO, das i. d. R. zum raschen/sofortigen Tod des:der Patient:in führt, einer sorgfältigen Vorbereitung und ausführlicher Gespräche innerhalb des Teams und mit den An- und Zugehörigen. In der Praxis hilft es zu betonen, dass eine nicht (mehr) indizierte Therapie, die nur mehr das Sterben hinauszögert und Leiden verlängert, beendet werden muss, um Sterben zuzulassen. Durch eine kritische Indikationsstellung für die ECMO-Anlage nach etablierten Kriterien [32, 33] können diese für alle Beteiligten maximal belastenden TZÄ vermieden werden.

### Flüssigkeits- und Ernährungstherapie

Die therapeutische Verabreichung von Flüssigkeit und Ernährung ist, wie alle Therapiemaßnahmen, an eine medizinische Indikation gebunden. Strafrechtliche Konsequenzen ergeben sich einerseits dann, wenn Flüssigkeit oder Ernährung trotz medizinischer Indikation verweigert werden, oder aber die Verabreichung gegen den Willen des:der einwilligungsfähigen Patient:in (Zwangsernährung) erfolgt.

Prinzipiell sind zwei Arten der Flüssigkeits- und Nahrungszufuhr zu unterscheiden: Bei der *pflegerischen Flüssigkeits- und Nahrungszufuhr* nimmt der:die Patient:in die angebotene Flüssigkeit/Nahrung freiwillig oral auf – entweder selbstständig oder mittels Handreichung (bei eingeschränkter Mobilität). Diese Art der Flüssigkeits- und Nahrungsversorgung ist nahezu immer indiziert und entspricht der Befriedigung eines menschlichen Grundbedürfnisses. Bei einer „Comfort feeding only care“ stehen die 3 Kernbedingungen für die Patient:innen im Mittelpunkt: „connect with me“, „see who I am“, und „include me“ [34, 35]. Eine *medizinisch indizierte Flüssigkeits- und Ernährungstherapie* wird über Sonden (nasogastral, perkutane endoskopische Gastrostomie [PEG]), oder parenteral durchgeführt. Sie muss wie jede andere Therapie indiziert, d. h. von einem klar definierten Therapieziel getragen sein. Im Rahmen der CTC erlischt die Indikation für eine Flüssigkeits- und Ernährungstherapie, die auf die Aufrechterhaltung der Homöostase ausgerichtet ist, was beim sterbenden Menschen selbstverständlich kein Therapieziel mehr darstellt. Entsprechend besteht auch kein Risiko für strafrechtliche Konsequenzen (s. oben).

In der Sterbephase bestehen praktisch nie Probleme durch Hunger und selten durch Dehydratation; häufig aber entstehen schwerwiegende klinische Folgen durch „Überwässerung“ (periphere Ödeme mit Schmerzen, Lungenödem mit Atemnot, Übelkeit/Erbrechen etc.) infolge eines Organversagens. Trotzdem erhält die Mehrzahl der Patient:innen am Lebensende im Krankenhaus eine Infusionstherapie, während in Hospizeinrichtungen auf parenterale Hydratation fast völlig verzichtet wird. Bruera zeigte, dass kein Unterschied besteht in den 4 dehydratationstypischen Symptomen Fatigue, Myoklonien, Schläfrigkeit und Auftreten von Halluzinationen zwischen sterbenden Patient:innen, die 1Liter 0,9 %ige NaCl-Lösung/Tag oder nur 100 Milliliter 0,9 %ige NaCl-Lösung/Tag infundiert bekommen. Flüssigkeitsgabe hat in dieser Situation weder die genannten Symptome noch die Lebensqualität verbessert und hat auch zu keiner Lebensverlängerung geführt [36]. Ebenso findet sich beim Sterbenden keine Korrelation zwischen subjektivem Durstgefühl und der intravenös verabreichten Flüssigkeitsmenge, der Blutharnstoffkonzentration und der Natrium-Plasma-Konzentration [37]. Das subjektive Durstgefühl wird beim Sterbenden hauptsächlich durch trockene Schleimhäute verursacht, daher bewirkt die Zufuhr von befeuchteter Raumluft eine deutliche Symptomlinderung. Zusätzliche Befeuchtung der Lippen und des Rachenraums mit z. B. feuchten Wattestäbchen und/oder durch Mundpflege mit Pflegeölen ergänzt sinnvoll die symptomatische Therapie des Durstgefühls.

### Monitoring und Befunderhebung

Monitoring und Befunde sind wichtige und unverzichtbare Werkzeuge zur Steuerung einer Therapie und zur Beobachtung des klinischen Verlaufs. Sowohl invasives als auch nichtinvasives Monitoring ist aber belastend für Patient:innen, die durch Kabel und Schläuche immobilisiert und stark bewegungseingeschränkt sind. Blutdruckmanschetten, Sättigungssensoren, Temperatursonden, Gesichtsmasken etc. können unangenehme und schmerzhafte Sensationen hervorrufen. Nach der Änderung des Therapieziels auf CTC besteht keine Indikation mehr für routinemäßiges Monitoring oder Befunderhebung, da aus pathologischen Werten/Befunden keine Konsequenzen mehr erwachsen. Ob in der Sterbephase ein Basismonitoring fortgesetzt wird, liegt in der Einschätzung des behandelnden Teams. Jedenfalls ist aber darauf zu achten, dass Alarme bettseitig an allen Geräten unterdrückt sind.

Auf der Normalstation ist in der Sterbephase auch bei laufender Analgosedierung kein Monitoring notwendig. Umso wichtiger sind in dieser Phase die klinische Beurteilung des:der Patient:in mit regelmäßiger Beurteilung der Zeichen von Unbehagen (Schmerzen, Stress, Atemnot, s. oben) und die symptomorientierte Steigerung der Analgosedierungsdosis. Auf eine umfassende ärztliche und pflegerische Dokumentation ist so wie bei auf Heilung ausgerichteter Therapie zu achten.

### Medikamentöse Therapie, Drainagen etc.

#### Thromboseprophylaxe

Das National Institute for Health and Care Excellence (NHS) gibt in der Guideline zur Venösen Thromboembolie beim Erwachsenen im Absatz 1.7.2 eine klare Stellungnahme zur Thromboseprophylaxe bei sterbenden Patient:innen ab: „Do not offer VTE prophylaxis to people in the last days of life“ [38].

#### Stressulkusprophylaxe

Da eine schwere Blutungskomplikation sowohl für den:die Patient:in als auch für das behandelnde Team und die An- und Zugehörigen äußerst belastend ist, sollte v. a. bei blutungsgefährdeten Patient:innen eine laufende Stressulkusprophylaxe weitergegeben werden.

#### Vasopressoren, Inotropika

Im Rahmen der Umstellung auf CTC ist die Therapie mit Vasopressoren und Inotropika zu beenden.

#### Antibiotika, Diuretika, Antiemetika, Laxanzien, Vitamine etc.

Im Rahmen der CTC sind alle Medikamente abzusetzen, die nicht dazu beitragen, die Symptomlast des:der Sterbenden zu reduzieren.

#### Umgang mit Drainagen, Magensonde etc.

Im Sinne eines friedlichen Sterbens sind liegende Drainagen nicht zu entfernen, da der:die Patient:in zu bluten beginnen könnte oder eine Leckage entsteht, was u. U. pflegerisch aufwendig zu versorgen ist oder sogar eine chirurgische Versorgung notwendig macht. Da Übelkeit und/oder Erbrechen häufige Symptome am Lebensende darstellen, wird die Magensonde zur Drainage von im Magen vorhandener Flüssigkeit zumeist auch in der Sterbephase belassen. Die (par)enterale Ernährung sollte zeitgerecht beendet werden (s. oben). Je nach Ursache wird eine zusätzliche medikamentöse Therapie belassen (s. oben) oder auch etabliert [39].

### Pflege im Rahmen der CTC

Die Pflege in der Sterbephase sollte vom Gedanken des Wohltuns getragen sein: Alles, was gut tut, sollte gemacht werden. Auf alles, was unangenehm ist, sollte verzichtet werden (z. B. Lagern nur mehr, wenn es dem:der Patient:in zugutekommt). Die Pflegemaßnahmen sollten im Sinne einer maximalen Symptomlinderung an die Bedürfnisse der Patient:innen angepasst werden. Wichtig ist z. B. das regelmäßige Befeuchten von Schleimhäuten, um Austrocknen zu verhindern (evtl. Verwenden von Duftölen). In der Betriebsamkeit einer Intensivstation ist die Umstellung auf eine Atmosphäre der Ruhe und des Loslassens oft nicht einfach zu bewerkstelligen. Das Gestalten einer „Insel der Besinnung“ (Paravents und Stühle aufstellen; alle An- und Zugehörigen gemeinsam ans Bett lassen; Getränke, Taschentücher bereitstellen; Duftlampe, Salzsteinleuchte aufstellen etc.) hilft dabei. Auf Wunsch des:der Patient:in und/oder der An- und Zugehörigen sollte eine spirituelle Begleitung gewährleistet werden. Oberstes Ziel ist es, den:die sterbende Patient:in zu umsorgen, mitfühlend zu begleiten und nicht allein zu lassen. Dies spendet Wohlbefinden und Trost für den:die Patient:in, für die An- und Zugehörigen und – nicht zuletzt – für das behandelnde Team.

### An- und Zugehörige

Wird ein:e Patient:in auf einer Intensivstation aufgenommen, so stellt dies eine Krise im Leben von An- und Zugehörigen dar. Es ist eine Zeit der Belastung und des Gefährdetseins. Sie wird erlebt als einschneidendes, katastrophales Ereignis, begleitet von der Angst des drohenden Verlusts, und bedarf der multidisziplinären Unterstützung (Ärzt:innen, Pflegende, Psycholog:innen, Seelsorger:innen, …) [40].

Die Entscheidung für CTC bedeutet nicht nur Begleitung sterbender Intensivpatient:innen, sondern auch Begleitung nahestehender Menschen in der ersten Phase von Schmerz, Abschied und Trauer. Diese erste Phase braucht Zeit für ein intensives Zusammensein [41]. Zu diesem Zweck ist es notwendig, unter Akzeptanz aller Beteiligten, die oft noch sehr starren Besuchszeiten aufzuheben.

In der unmittelbaren Sterbephase öffnet CTC einen Raum für die intimen Begegnungen der An- und Zugehörigen mit dem sterbenden Menschen. Angehörige sollten zur Nähe ermutigt werden.

Für die An- und Zugehörigen ist es hilfreich, wenn aktiv kommuniziert wird, dass dem geliebten Menschen effektive Linderung von Schmerz und anderen Symptomen zukommt [42]. Die Aspekte der Palliativmedizin mit Symptomkontrolle, Zentrierung auf die Bedürfnisse des:der Sterbenden, Beachtung der sozialen, spirituellen und psychischen Dimensionen des Sterbeprozesses [43] sowie die spürbare Akzeptanz des nun nahenden Todes können entlastend auf die An- und Zugehörigen wirken.

Es konnte gezeigt werden, dass ein strukturiertes, geplantes Vorgehen im Rahmen der Sterbebegleitung mit proaktiven Gesprächen zu definierten Zeiten zum geringeren Auftreten von „Post-Intensive-Care-Syndrome Family“ (PICS-F) und auch einer geringeren Ausprägung von Symptomen der Angst führt. Bewährt haben sich Gespräche im Rahmen einer Familienkonferenz 1) vor dem Patient:innenbesuch zur Vorbereitung der An- und Zugehörigen auf den bevorstehenden Tod, 2) während des Besuches, um aktive Unterstützung anzubieten, und 3) nach dem Tod, um zu kondolieren [44].

In der Kommunikation mit An- und Zugehörigen soll auf Euphemismen verzichtet werden; die Kommunikation soll klar, verständlich, ehrlich und empathisch sein [45]. Eine psychologische, salutogenetische Begleitung zur Unterstützung in Krisensituationen, um An- und Zugehörige bestmöglich zu stabilisieren und einer Dekompensation vorzubeugen, ist empfehlenswert [46].

### Nach dem Versterben

Für An- und Zugehörige endet die schwierige Zeit nicht mit dem Versterben; sie ist geprägt von der persönlichen Auseinandersetzung mit dem Thema Abschied, Tod und Trauer. Die persönliche Beziehung lebt weiter, und es braucht die Beachtung der letzten Wünsche und die Würdigung des:der Patient:in [47]. Abschied braucht Zeit, Ruhe und Raum. Ideal ist ein ruhiger Verabschiedungsraum, der den spirituellen Bedürfnissen der Verstorbenen angepasst werden kann [48]. So wie das Ende des Lebens in einem würdigen Rahmen erfolgen soll, soll es auch möglich sein, dass An- und Zugehörige sich in aller gebotenen Würde von dem:der Verstorbenen verabschieden.

Von Hinterbliebenen wurde mitgeteilt, dass sie den abrupten Abbruch der Beziehungen im Todesfall nach z. T. lange währenden Intensivstationsaufenthalten als sehr belastend empfanden [38]. Eine proaktive Trauerbegleitung kann hier nachhaltig Angstzustände, Depressionen, posttraumatische Belastungsstörungen und komplizierte Trauer für Familien mildern [49].

Trauerbegleitung, einschließlich Informationen über Selbsthilfegruppen, Zusendung einer Beileidskarte einige Wochen nach dem Todesfall und eines Folgegesprächs, sind sehr effektive Maßnahmen [50]. Auch das Aushändigen von Informationsbroschüren rund um den Tod (Worte der Trauer, Informationen, was nun mit dem Körper geschieht, welche Möglichkeiten der weiteren Verabschiedung es gibt, welche (amtlichen) Wege nun anstehen, Informationen zu Trauerarbeit, Begleitungs- und Beratungsgruppen, …) geben Hinterbliebenen das Gefühl, dass sie in ihrer Trauer ernst- und wahrgenommen werden [51].

## Zusammenfassung

Die Überprüfung des Therapiezieles und die damit verbundene kritische Frage, ob es noch eine Indikation für die Fortführung laufender Therapien gibt, sollte bei schwer erkrankten Patient:innen regelmäßig geübte Praxis im Behandlungsteam sein. Eine TZÄ mit zeitgerechter Umstellung auf CTC ermöglicht eine gute und menschliche Betreuung schwerst kranker Patient:innen und ihrer An- und Zugehörigen am Lebensende und die Wertschätzung ihres bisherigen Lebens mit der Möglichkeit positiver Erfahrungen bis zuletzt.
